# The Association Between Patient Satisfaction and Mode of Visit (Telemedicine Versus In-Person) in a Large Orthopaedic Practice During the COVID-19 Pandemic Lockdown: A Retrospective Study

**DOI:** 10.5435/JAAOSGlobal-D-21-00046

**Published:** 2021-09-21

**Authors:** Leslie J. Bisson, Melissa A. Kluczynski, Carter M. Lindborg, Michael A. Rauh, Matthew J. DiPaola, Mohammad N. Haider, Sonja Pavlesen

**Affiliations:** From the Jacobs School of Medicine & Biomedical Sciences, University at Buffalo, Buffalo, NY.

## Abstract

**Background:**

During the novel coronavirus disease 2019 (COVID-19) pandemic, telemedicine was rapidly adopted to provide continued, efficient, and safe medical care. Little is known about patient satisfaction with telemedicine in orthopedics or the factors associated with selection of telemedicine versus face-to-face care. Thus, we examined (1) the association between patient satisfaction and mode of visit (telemedicine versus in-person) and (2) predictors of patient satisfaction in a large orthopedic practice during the onset of the pandemic.

**Methods:**

We conducted a retrospective cohort study of in-person and telemedicine visits within a large, university-affiliated orthopaedic practice between March 2020 and April 2020 during the onset of the COVID-19 pandemic. Patients who completed a patient satisfaction survey were included. Demographic and other office visit (eg, type of provider and type of visit) data were collected. A Patient Satisfaction Aggregate (PSA, range 0 to 1) score was calculated by taking the average of five patient satisfaction questions. Linear regression was used to examine (1) the association between PSA score and mode of visit and (2) predictors of PSA score.

**Results:**

A total of 2,049 of 6,515 patient satisfaction surveys were completed and included for analysis, of which 748 had telemedicine visits and 1,301 had in-person visits. No association was found between PSA score and mode of visit with and without adjustment for duration of patient-physician relationship, appointment type (new versus follow-up), provider type (physician versus nonphysician), and provider subspecialty (β_unadjusted_ = 0.004 [SE = 0.01], *P* = 0.44; β_adjusted_ = 0.001 [SE = 0.01], *P* = 0.92). Predictors of increased PSA score were White race (*P* = 0.001), >1 year relationship with provider (*P*_1-3 years_ = 0.01, *P*_3-5 years_ = 0.04, and *P*_5+ years_ = 0.002), physician provider (*P* = 0.004), and foot/ankle provider (*P* = 0.04), whereas predictors of decreased PSA score were oncology provider (*P* = 0.02) and spine provider (*P* = 0.001).

**Conclusion:**

We found no association between PSA score and mode of visit. Predictors of PSA score included race, duration of patient-physician relationship, provider type, and provider subspecialty.

The effect of the novel coronavirus disease 2019 (COVID-19) pandemic has been far-reaching in the field of orthopaedics.^1^ In an attempt to control the spread of the virus yet allow for continued ambulatory health care, telemedicine visits increased following the Centers for Medicare and Medicaid Services waiver regarding telehealth and recommendations for delaying all nonessential medical procedures in March 2020.^2^ Many practices rapidly adopted telehealth services in orthopaedics,^3^ aided by the federal Coronavirus Preparedness and Response Supplemental Appropriations Act that allowed Medicare to pay for visits conducted through telehealth,^2^ encouraging safe and appropriate medical care.

Telemedicine is a telecommunication system that enables providers to care for their patients virtually.^2^ Benefits of telemedicine include direct access to care, convenience, ease of use, patient choice, on-demand services, cost-effectiveness, and efficiency.^4^ However, challenges with telemedicine may include the inability to perform a full physical assessment, technological difficulties, limited patient-provider interaction, and reduced access for certain demographics (eg, patients with low socioeconomic status, disabilities, and the elderly).^5,6^

Historically, orthopaedics has not often used the full extent of telemedicine services,^7^ and previous studies were limited to remote consultations,^8^ postoperative follow-up care,^9^ and/or rehabilitation.^10^ Rizzi et al.^3^ found that for 78.4% of 612 orthopedic telehealth encounters, surgeons felt that the telehealth encounter successfully replaced an in-person visit. The authors also reported that 95% of patients felt their surgeon was sensitive to their needs and appropriately addressed their concerns, and 93% of patients would do the telemedicine encounter again. In a randomized controlled trial predating the COVID-19 pandemic, Kane et al.^9^ evaluated the application of telemedicine for the surgical follow-up in 66 patients after rotator cuff surgery. The authors found similar overall pain and satisfaction scores between telemedicine and in-office visits, and both providers and patients found telemedicine visits to be less time-consuming than in-office visits. Owing to an increase in demand and guidance resulting from COVID-19, recent publications have presented the effectiveness of using telemedicine services in orthopaedics^11^ including descriptive musculoskeletal assessment protocols to use during virtual examinations.^12,13^

To our knowledge, no study has examined whether patient satisfaction differs between telemedicine and in-person visits in a large cohort of orthopedic patients. In an effort to better understand the adoption and acceptance of telemedicine in orthopaedics during the COVID-19 pandemic, we performed a retrospective comparative study to examine the association between patient satisfaction and mode of visit (in-person versus telemedicine) within a large, university-affiliated orthopaedic practice between March 2020 and April 2020. Our primary aim was to examine the association between patient satisfaction and mode of visit (telemedicine versus in-person) with and without adjustment for patient demographics and visit characteristics (eg, type of provider). Our secondary aim was to identify predictors of patient satisfaction. We hypothesized that there would be no association between patient satisfaction and mode of visit and that multiple demographic and visit characteristics would predict patient satisfaction.

## Methods

### Study Design and Participants

Our university's institutional review board approved this retrospective comparative study. We identified patients of all ages (children and adults) who were seen through in-person or telemedicine by one of the 51 providers (23 medical doctors, 25 physician's assistants, and 3 nurse practitioners) in our practice during the onset of the COVID-19 pandemic (March 1, 2020 to April 30, 2020) and completed our institution's standard Patient Satisfaction Survey within 2 weeks of their visit. A complete list of patients seen during the inclusion period was screened for duplicate patient medical record numbers. If patients were seen more than once during the inclusion period, the earlier of the two visits and associated satisfaction survey were included in this study.

### Demographic and Clinical Data Collection

The following demographic variables were extracted from electronic medical records and were verified by three trained research assistants: age, sex (male or female), race (White, Black, other, more than 1, or did not specify), median household income by zip code, and type of insurance (private, Medicare, other, no fault/worker's compensation, or uninsured). The median income for each zip code was obtained from New York State Income Statistics, US Census Bureau.^14^ Clinical variables included duration of patient-physician relationship (first visit, less than 6 months, 6 to 12 months, 1 to 3 years, 3 to 5 years, and 5+ years), type of visit (new or follow-up), provider type (medical doctors or physician's assistants/nurse practitioners), and provider subspecialty (foot/ankle, joint surgery, oncology, urgent care/trauma, pediatrics, physical medicine and rehabilitation, primary care sports medicine, spine surgery, sports surgery, and upper extremity). After data collection was complete, an independent research associate inspected the data in 10 random blocks of 20 for accuracy.

### Patient Satisfaction Data

The primary outcome measure was patient satisfaction obtained from our institution's Patient Satisfaction Survey. This survey is emailed to patients after their clinical visit as part of standard clinical practice. To avoid overwhelming patients with emails, there is a minimum of 30 days between sending surveys if the patient had multiple visits within that timeframe (i.e., patients are eligible to receive the survey if it had been at least 30 days since a previous survey was sent or if they were new to the practice and never received a survey). Patient satisfaction data were stored in our institution's Outcomes Based Electronic Research Database, a Health Insurance Portability and Accountability Act (HIPAA)-compliant online data capture software program and was subsequently extracted from this database for analysis. The satisfaction survey assessed the following: (1) duration of provider-patient relationship (first appointment, <6 months, 6 to 12 months, 1 to 3 years, 3 to 5 years, and >5 years); (2) patient feels their physician cares about them (yes or no); (3) provider's explanations were easy to understand (5-point scale, strongly disagree to strongly agree); (4) provider spent enough time with me (5-point scale, strongly disagree to strongly agree); (5) overall service received from physician (5-point scale, poor to excellent); (6) likelihood of recommending to family, friend, and co-workers (5-point scale, strongly disagree to strongly agree); (7) overall service received from call center, physical therapy, physician's staff, surgery scheduler, and billing personnel (5-point scale, poor to excellent); (8) where they heard about the practice; and (9) any general comments. The Patient Satisfaction Aggregate (PSA) score was estimated by taking the average of the five questions stated above with 5-point interval scales and transformed to a 0 to 1 continuous scale. Responses to the two questions regarding “duration of patient-provider relationship” and “patient feels their physician cares about them” were not part of the aggregate score.

### Statistical Analysis

Descriptive statistics were calculated for patient demographics, visit characteristics, and PSA score both for the overall study sample and stratified by mode of visit (telemedicine versus in-person). Group comparisons were made with *t*-tests for continuous data and chi-square or Fisher's exact test for categorical data. We examined the univariate association between PSA score and all demographic and visit characteristic variables using analysis of variance for categorical data and the Pearson correlation for continuous data. When analysis of variance demonstrated statistical significance, the Tukey procedure was used for additional post hoc testing. Univariate and multivariate linear regression was used to examine the association between PSA score and mode of visit. The multivariate model was adjusted for duration of patient-physician relationship, appointment type, provider type, and provider subspecialty because these variables were statistically significantly associated with both mode of visit and PSA in the univariate analyses described above. Next, stepwise linear regression was used to identify the most predictive multivariate model for the PSA score. The following variables were included in the stepwise model: sex, race, mode of visit, duration of patient-physician relationship, type of visit, provider type, provider subspecialty, and insurance type. The predictive variables that were identified as being statistically significant through stepwise regression were then added to a multivariate linear regression model to estimate the β coefficients, *P*-values, and R^2^ for the overall predictive model. Finally, we conducted a sensitivity analysis to examine whether the study results remained the same when restricted to only follow-up visits because established patients may be more receptive to telemedicine visits than new patients. Statistical analyses were performed using SAS version 9.4 (SAS Institute).

## Results

**Figure 1:**
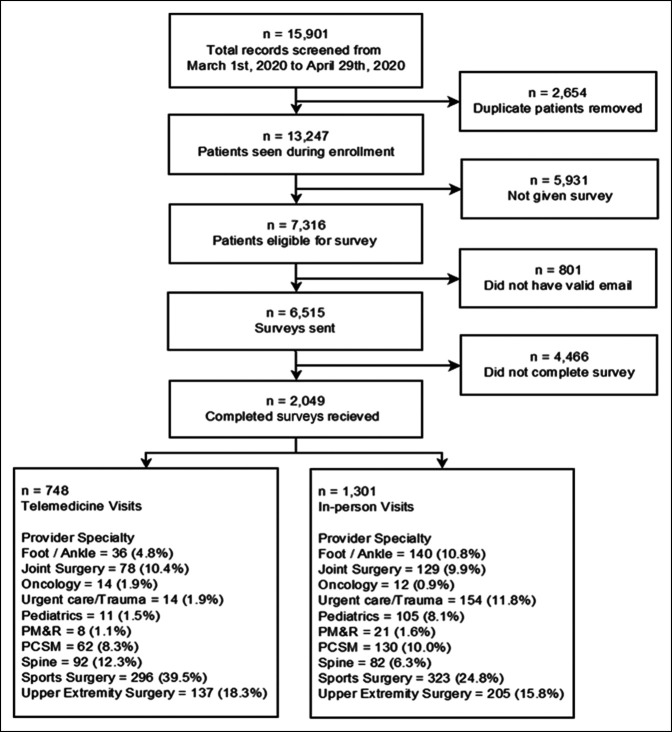
Sample flowchart.

**Figure 2:**
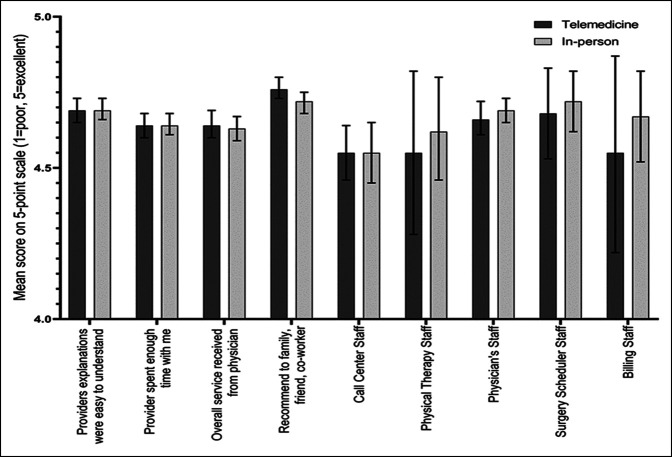
Graph showing the mean patient satisfaction survey scores by mode of visit. Scores based on a five-point Likert scale with 95% confidence limits (1 = strongly disagree to 5 = strongly agree or 1 = poor to 5 = excellent). No significant differences between modes of visit were observed for explanation (*P* = 0.22), spending enough time (*P* = 0.23), overall service from physician (*P* = 0.28), recommend to others (*P* = 0.59), call center (*P* = 0.49), physical therapy (*P* = 0.75), physician staff (*P* = 0.16), or billing staff (*P* = 0.23).

**Figure 3:**
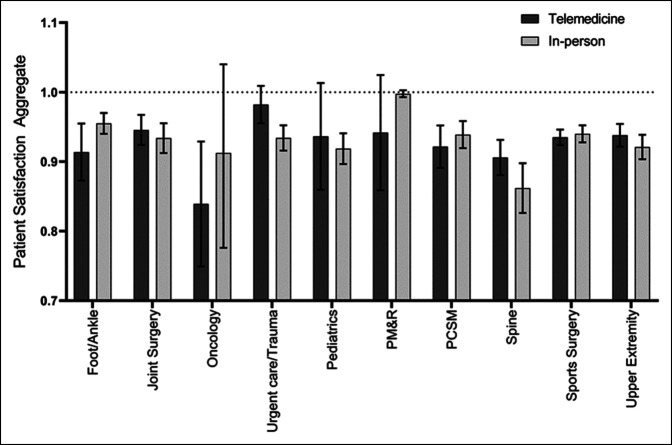
Graph showing the patient satisfaction aggregate score stratified by mode of visit and provider subspecialty. Patient Satisfaction Aggregate score transformed to a 0 to 1 scale. No significant differences, after post hoc correction, were observed between modes of visit for foot/ankle (*P* = 0.05), joint surgery (*P* = 0.77), oncology (*P* = 0.05), urgent care/trauma (*P* = 0.07), pediatrics (*P* = 0.60), physical medicine and rehabilitation (PM&R, *P* = 0.17), primary care sports medicine (PCSM, *P* = 0.22), spine (*P* = 0.12), or upper extremity (*P* = 0.54).

A total of 13,247 patients were seen between March 1, 2020, and April 30, 2020, of which 6515 were emailed a patients satisfaction survey (the remainder were not eligible because it had been ≤30 days since their last survey was sent or the patient refused to provide an e-mail address, Figure 1). The survey was completed by 2,049 patients (748 telemedicine visits and 1,301 in-person visits) who were included in the final analysis (Figures 2 and 3).

Table 1 summarizes demographics and visit characteristics stratified by mode of visit and overall. The mean age of the overall study sample was 53.37 ± 18.13 years, and most of the sample was female (55.9%) and White (92.7%). When stratified by mode of visit, there were statistically significant differences for sex (*P* = 0.02), race (*P* = 0.0002), duration of patient-physician relationship (*P* < 0.0001), median household income (*P* = 0.03), type of visit (*P* < 0.001), provider type (*P* < 0.001), provider subspecialty (*P* < 0.0001), and insurance type (*P* < 0.0001). The overall mean PSA score was 0.92 ± 0.13. As given in Table 2, there was a statistically significant difference in mean PSA for categories of race (*P* < 0.0001), duration of patient-physician relationship (*P* = 0.0003), type of visit (*P* = 0.03), provider type (*P* = 0.01), and provider subspecialty (*P* < 0.0001). However, mean PSA did not differ by sex (*P* = 0.17), mode of visit (*P* = 0.44), and insurance type (*P* = 0.30). A statistically significant (albeit weak) correlation was found between age and PSA score (R = 0.05, *P* = 0.02), but not between median household income and PSA score (R = 0.03, *P* = 0.16, Table 3).

**Table 1:** Demographics and Visit Characteristics for the Total Sample and Stratified by Mode of Visit

Demographics and Visit Characteristics	Telemedicine Visits (N = 748)	In-person Visits (N = 1,301)	Total (N = 2,049)	*P* Value^a^
Age (yr), mean ± SD	54.36 ± 15.6	52.80 ± 19.4	53.37 ± 18.13	0.05
Sex, N (%)				0.02
Male	355 (47.5%)	549 (42.2%)	904 (44.1%)	
Female	393 (53.3%)	752 (57.8%)	1,145 (55.9%)	
Race, N (%)				0.0002
White/Caucasian	689 (92.1%)	1,211 (93.1%)	1,900 (92.7%)	
Black	22 (2.9%)	40 (3.1%)	62 (3.0%)	
Other	13 (1.7%)	20 (1.5%)	33 (1.6%)	
More than one	4 (0.5%)	6 (0.5%)	10 (0.5%)	
Did not specify	20 (2.7%)	24 (1.8%)	44 (2.2%)	
Duration of patient-physician relationship				<0.0001
First visit	116 (15.5%)	655 (50.4%)	771 (37.6%)	
Less than 6 months	236 (31.6%)	199 (15.3%)	435 (21.2%)	
6-12 months	148 (19.8%)	106 (8.2%)	254 (12.4%)	
1-3 years	150 (20.1%)	160 (12.3%)	310 (15.1%)	
3-5 years	46 (6.2%)	67 (5.2%)	113 (5.5%)	
5+ years	52 (7.0%)	114 (8.8%)	166 (8.1%)	
Median household income by zip code (USD), mean ± SD^b^	65,595 ± 18,681	67,520 ± 20,187	66,818 ± 19,669	0.03
Type of visit, N (%)				<0.001
New visit	40 (5.4%)	817 (62.8%)	857 (41.8%)	
Follow-up visit	708 (94.7%)	484 (37.2%)	1,192 (58.2%)	
Provider type, N (%)				< 0.001
Physician	547 (73.1)	769 (59.1)	1,316 (64.2%)	
Nonphysician	201 (26.9)	532 (40.9)	733 (35.8%)	
Provider subspecialty, N (%)				<0.0001
Foot/ankle	36 (4.8%)	140 (10.8%)	176 (8.6%)	
Joint surgery	78 (10.4%)	129 (9.9%)	207 (10.1%)	
Oncology	14 (1.9%)	12 (0.9%)	26 (1.3%)	
Urgent care/trauma	14 (1.9%)	154 (11.8%)	168 (8.2%)	
Pediatrics	11 (1.5%)	105 (8.1%)	116 (5.7%)	
Physical medicine and rehabilitation	8 (1.1%)	21 (1.6%)	29 (1.4%)	
Primary care sports medicine	62 (8.3%)	130 (10.0%)	192 (9.4%)	
Spine	92 (12.3%)	82 (6.3%)	174 (8.5%)	
Sports surgery	296 (39.6%)	323 (24.8%)	619 (30.2%)	
Upper extremity	137 (18.3%)	205 (15.8%)	342 (16.7%)	
Insurance type, N (%)				<0.0001
Private	365 (48.8%)	697 (53.6%)	1,062 (51.8%)	
Medicare	78 (10.4%)	134 (10.3%)	212 (10.4%)	
Other	172 (23.0%)	341 (26.2%)	513 (25.0%)	
No fault/Worker's compensation	133 (17.8%)	126 (9.7%)	259 (12.6%)	
Uninsured	0 (0%)	3 (0.2%)	3 (0.2%)	

a *P*-values represent the comparison between telemedicine and in-person visits. T-tests were used to calculate *P*-values for continuous variables and χ^2^ tests for categorical data.

b Median income is missing for 11 patients.

**Table 2:** Mean Patient Satisfaction Aggregate (PSA) Score by Categorical Variables

Categorical Variables	N	PSA Score	*P* Value^a^
		Mean ± SD	
Sex			0.17
Male	904	0.93 ± 0.12	
Female	1,145	0.92 ± 0.13	
Race			<0.0001^b^
White/Caucasian	1,900	0.93 ± 0.12	
Black	62	0.86 ± 0.17	
Other	33	0.91 ± 0.12	
More than one	10	0.85 ± 0.25	
Did not specify	44	0.86 ± 0.18	
Duration of patient-physician relationship			0.0003^c^
First visit	771	0.91 ± 0.14	
Less than 6 months	435	0.92 ± 0.12	
6-12 months	254	0.92 ± 0.11	
1-3 years	310	0.94 ± 0.12	
3-5 years	113	0.94 ± 0.10	
5+ years	166	0.96 ± 0.10	
Type of visit			0.03
New visit	857	0.92 ± 0.14	
Follow-up visit	1,192	0.93 ± 0.12	
Mode of visit			0.44
Telemedicine	748	0.93 ± 0.11	
In-person	1,301	0.92 ± 0.13	
Provider type			0.01
Physician	1,316	0.93 ± 0.12	
Nonphysician	733	0.91 ± 0.14	
Provider subspecialty			<0.0001^d^
Foot/ankle	176	0.94 ± 0.12	
Joint surgery	207	0.93 ± 0.12	
Oncology	26	0.86 ± 0.21	
Urgent care/trauma	168	0.93 ± 0.12	
Pediatrics	116	0.91 ± 0.12	
Physical medicine and rehabilitation	27	0.98 ± 0.07	
Primary care sports medicine	192	0.93 ± 0.13	
Spine	174	0.88 ± 0.16	
Sports surgery	619	0.93 ± 0.11	
Upper extremity	342	0.92 ± 0.12	
Insurance type			0.30
Private	1,062	0.92 ± 0.13	
Medicare	212	0.93 ± 0.12	
Other	513	0.92 ± 0.13	
No fault/Worker's compensation	259	0.93 ± 0.12	
Uninsured	3	1.00 ± 0	

a *P*-values were calculated with analysis of variance.

b There was a significant difference in PSA between (1) White patients and Black patients and (2) White patients and patients who did not specify race based on Tukey post hoc testing.

c There was a significant difference in PSA between (1) first visit and 5+ years, (2) first visit and 1 to 3 years, and (3) 6 to 12 months and 5+ years based on Tukey post hoc testing.

d There was a significant difference in PSA between (1) spine and foot/ankle, (2) spine and joint, (3) spine and urgent/trauma, (4) spine and physical medicine and rehabilitation, (5) spine and primary care sports medicine, (6) spine and upper extremity, and (7) oncology and physical medicine and rehabilitation based on Tukey post hoc testing.

**Table 3:** Pearson Correlations Between Patient Satisfaction Aggregate (PSA) Score and Continuous Variables

Continuous Variables	Total N = 2,049
Age in years	r = 0.05 (*P* = 0.02)
Median household income by zip code in USD^**a**^	r = 0.03 (*P* = 0.16)

a Median income is missing for five patients.

As shown in Table 4, both the unadjusted (β = 0.004 [SE = 0.01], *P* = 0.44) and adjusted (β = 0.001 [SE = 0.01], *P* = 0.92) linear association between PSA score and mode of visit were not statistically significant. The final multivariate predictive model explained approximately 5% of the variance in PSA score (Table 5). Race (2%) and provider subspecialty (2%) explained the most variance, followed by duration of patient-provider relationship (1%) and provider type (0.4%). Predictors of increased PSA score were White race (*P* = 0.001), >1 year relationship with provider (*P*_1-3 years_ = 0.01, *P*_3-5 years_ = 0.04, and *P*_5+ years_ = 0.002), physician provider (*P* = 0.004), and foot/ankle provider (*P* = 0.04). Predictors of decreased PSA score were oncology provider (*P* = 0.02) and spine provider (*P* = 0.001). None of the other predictors were associated with the PSA score.

**Table 4:** Unadjusted and Adjusted Linear Regression Models Examining the Association Between Patient Satisfaction Aggregate (PSA) Score and Mode of Visit (Telemedicine Versus In-Person)

Mode of visit	N	Unadjusted β (SE)	Unadjusted *P*-Value	Adjusted β (SE)^a^	Adjusted *P*-Value^a^
In-person visit	748	Referent	—	Referent	—
Telemedicine visit	1,301	0.004 (0.01)	0.44	0.001 (0.01)	0.92

a Adjusted for duration of patient-physician relationship, appointment type, provider type, and provider subspecialty.

**Table 5:** Multivariate Predictive Model for Patient Satisfaction Aggregate (PSA) Score

Predictor	Unadjusted β (SE)	Unadjusted *P*-Value	Adjusted β (SE)^a^	Adjusted P-Value^a^	Partial R^2^	Model R^2^
Race					0.02	0.04
White/Caucasian	0.07 (0.02)	0.001	0.06 (0.02)	0.001		
Black	0.0003 (0.02)	0.99	−0.01 (0.02)	0.79		
Other	0.05 (0.03)	0.10	0.04 (0.03)	0.16		
More than one	−0.01 (0.04)	0.86	−0.01 (0.04)	0.81		
Did not specify	Referent	—	Referent	—		
Duration of patient-physician relationship					0.01	0.05
First visit	Referent	—	Referent	—		
Less than 6 months	0.01 (0.01)	0.10	0.01 (0.01)	0.06		
6-12 months	0.01 (0.01)	0.43	0.01 (0.01)	0.47		
1-3 years	0.03 (0.01)	0.003	0.02 (0.01)	0.01		
3-5 years	0.03 (0.01)	0.02	0.03 (0.01)	0.04		
5+ years	0.04 (0.01)	<0.0001	0.03 (0.01)	0.002		
Provider type, N (%)					0.004	0.04
Physician	0.02 (0.01)	0.01	0.02 (0.01)	0.004		
Nonphysician	Referent	—	Referent	—		
Provider subspecialty, N (%)					0.02	0.02
Foot/ankle	0.01 (0.01)	0.22	0.02 (0.01)	0.04		
Joint surgery	0.01 (0.01)	0.51	0.01 (0.01)	0.24		
Oncology	−0.06 (0.03)	0.01	−0.06 (0.03)	0.02		
Urgent care/trauma	0.003 (0.01)	0.77	0.01 (0.01)	0.30		
Pediatrics	−0.01 (0.01)	0.33	−0.01 (0.01)	0.30		
Physical medicine and rehabilitation	0.06 (0.02)	0.02	0.03 (0.03)	0.19		
Primary care sports medicine	0.005 (0.01)	0.65	0.002 (0.01)	0.83		
Spine	−0.05 (0.01)	<0.0001	−0.04 (0.01)	0.001		
Sports surgery	0.008 (0.008)	0.32	0.01 (0.01)	0.39		
Upper extremity	Referent	—	Referent	—		
Fully adjusted model R^2^						0.05

a Adjusted for all other covariates in the model.

As a sensitivity analysis, we repeated all analyses for follow-up visits only (N = 1,192). The statistically significant results largely remained the same (Appendix Tables 1–5, http://links.lww.com/JG9/A147), with the following exceptions described here. No longer a statistically significant difference was observed in sex (*P* = 0.27) and insurance type (*P* = 0.09) when stratified by mode of visit (Appendix Table 1, http://links.lww.com/JG9/A147). The results of the multivariate predictive model remained the same, except sex was added to the final model based on the results of stepwise regression (Appendix Table 5, http://links.lww.com/JG9/A147). Foot/ankle providers (*P* = 0.80) and spine providers (*P* = 0.05) were no longer statistically significant predictors of PSA score.

## Discussion

Our large, retrospective study comparing patient satisfaction between in-person and telemedicine visits during the beginning of the COVID-19 lockdown found no association between patient satisfaction and mode of visit. Our predictive model explained only 5% of the variance in PSA score. Predictors of increased PSA score were White race, >1 year relationship with provider, physician provider, and foot/ankle provider, and predictors of decreased PSA score were oncology provider and spine provider.

Our findings are consistent with the study of Rizzi et al.^3^ which found no difference in patient satisfaction between telemedicine and in-person orthopedic encounters during the COVID-19 pandemic. Although telemedicine is not a replacement for all types of patient encounters, it does seem to be an acceptable form of consultation for patients. Moreover, we found that patient satisfaction was higher for White patients, >1 year relationship with provider, those seen by a physician, and those seen by a foot/ankle provider. Only about 7% of our study sample was non-White, which increases the chance of a type I error when exploring racial differences, so this finding should be interpreted with caution. Patients tend to prefer seeing a surgeon for certain types of visits, including new patient visits and surgical consultation, as opposed to a midlevel provider.^15,16^ Increased confidence in physician providers may have subsequently contributed to increased patient satisfaction for these encounters. Furthermore, 64% of visits were with physician providers. Our retrospective study design precluded determining the reason for this; however, telehealth visits are often less time-consuming and may allow for increased patient volume.^9^ Previous studies^17-19^ have already investigated the cost-benefit relationship between telemedicine and in-person visits and have concluded that telemedicine is usually effective, often requires less time and resources, and is expected to become a part of standard clinical care.^20^ However, in contrast to our findings, a previous systematic review found no notable differences in patient satisfaction between physicians and nonphysician providers.^21^ Predictors of decreased PSA score were oncology provider and spine provider. This may reflect the reason for patients visit, such as the severity of their injury or condition or the degree to which their daily function has been affected.^22,23^

A higher proportion of in-person visits in our sample had new appointments compared with telemedicine visits; however, it is unclear from our retrospective design whether this was based on patient or provider preference. Perhaps patients felt more comfortable scheduling a telehealth encounter if they already had an established relationship with their provider for their specific issue.^24,25^ Alternatively, physicians may have felt that conducting a new appointment encounter through telemedicine may not provide sufficient interaction to achieve an adequate assessment and treatment.^17^ To examine this further, we performed a sensitivity analysis including only follow-up visits and the results essentially remained the same suggesting that type of visit did not affect the relationship between PSA score and mode of visit in our study.

This study is not without limitations, including its retrospective design, which precluded us from determining causality. In addition, the validity of self-reported patient satisfaction surveys is unknown. However, these surveys are routinely used as part of standard clinic protocols, increasing the external validity of our findings. Although we observed a high rate of nonresponse for patient satisfaction (68.5%), it is comparable with that of previous studies.^26-28^ In addition, our study did not consider all of the factors that may have influenced the association between patient satisfaction and mode of visit, such as place of residence, education, medical comorbidities, pain, and function. Furthermore, our multivariate model was only able to account for 5% of the variance in PSA score. This finding also suggests that other factors not measured by this study are affecting the PSA score. Finally, we captured data from the onset of the COVID-19 pandemic, which likely affected patients' emotional and behavioral characteristics increasing anxiety and feeling of functional impairment and may have subsequently affected their response resulting in response bias.

## Conclusion

We found no association between PSA score and mode of visit. Predictors of PSA score included race, duration of patient-physician relationship, provider type, and provider subspecialty (http://links.lww.com/JG9/A147).
